# Aromatase Inhibitor Monotherapy to Augment Height in Boys: Does It Work and Is It Safe?

**DOI:** 10.1210/jendso/bvae196

**Published:** 2024-11-06

**Authors:** Mitchell E Geffner

**Affiliations:** Children's Hospital Los Angeles, Keck School of Medicine of the University of Southern California, Los Angeles, CA 90033, USA

**Keywords:** aromatase inhibitors, letrozole, anastrozole, height

There continues to be a drive toward medicalization of short stature (mostly in males), especially in the United States, leading to multiple pharmaceutical approaches in children and adolescents to augment growth, and also cosmetic limb-lengthening surgery in short adults. The medical treatments include broader use of growth hormone (GH) in both boys and girls, and pubertal manipulation in short or predicted-to-be short adolescent males with rapidly advancing puberty and bone ages (BAs). The latter includes gonadotropin-releasing hormone analogs to suppress testosterone (and ultimately estrogen) or aromatase inhibitors (AIs) to suppress conversion of androgens to estrogens, with estrogens the hormonal class in both sexes leading to epiphyseal fusion.

The recent paper by Zegarra et al [1] in the *Journal of the Endocrine Society* reports near-adult heights (NAHs) of 79 pubertal males with idiopathic short stature (ISS) > 10 years, BAs < 14 years, and predicted adult heights (PAHs) < 5th percentile or >10 cm below mid-parental height who were randomized to receive either anastrozole (1.0 mg) or letrozole (2.5 mg) daily for up to 3 years. Unfortunately, there was no contemporaneous control group. The authors found that there was an increase in PAH after 1 year in both groups, which, however, did not persist in either group after 2 to 3 years of treatment. Letrozole caused greater increases than anastrozole in testosterone, dihydrotestosterone, luteinizing hormone, and follicle-stimulating hormone levels and height velocity, and slower BA advancement, but no group differences in PAH (a mere 1.3-cm gain) vs baseline predictions. Thus, the authors concluded that efficacy of AIs as monotherapy for height augmentation in pubertal boys with ISS appears limited.

AIs have been widely used to augment height despite the fact that they are Food and Drug Administration–approved only as adjunctive therapy for postmenopausal women with estrogen-sensitive breast cancer. There have been no industry-sponsored or investigator-initiated, large-scale, prospective, randomized, blinded, long-term clinical trials of AI monotherapy to augment growth. Published data are limited and mixed in terms of efficacy due to small cohort sizes; non-blinding; and inclusion of patients with different chronological and BAs, as well as varying underlying causes of short stature (eg, GH deficiency, constitutional delay of growth and puberty, and ISS [itself with varying definitions]). Most studies have been retrospective, with varying follow-up periods and usually not until NAH or adult height; have lacked an adequate control group; and included subjects receiving concomitant treatments (eg, GH or testosterone) to which introduction of AIs appears to have an additive effect on height. A subanalysis as part of a 2015 Cochrane review suggested that AI monotherapy with letrozole also showed some short-term height benefit. This was confirmed in a more recent systematic review and meta-analysis (analyzing much of the same data) in 2021, which reported an improved PAH (by ∼5 cm) in those receiving AI monotherapy [2]. However, one of the earliest AI monotherapy cohorts which showed an initial increase in PAH failed to show any benefit when the subjects were followed to NAH [3].

What do we know about the side effects of AIs? Not much—or at least not enough! In the current report from Zegarra et al, frequent side effects were noted across both groups including vertebral abnormalities such as biconcave vertebral body, spondylolysis, irregular endplates both at baseline and after 2 years in 3 subjects, and statistically significant decreases in whole-body and lumbar bone density from baseline to 2 years in both groups. Additionally, anterior wedging was noted in one participant > 4 years after treatment initiation. These vertebral findings appear consistent with earlier reports suggesting that there may be an increased risk of mildly deformed vertebral bodies in boys treated with letrozole, but which usually self-remit when reassessed by magnetic resonance imaging [3]. Similarly, 5 subjects in the Zegarra et al study sustained sports-related elbow and wrist fractures, 1 incurred a hip avulsion fracture while playing soccer, and 3 had scoliosis. Both AI groups reported changes in behavior, hair loss, and acne possibly due to induced elevations in testosterone. However, without a control group, any connection to AIs cannot be established with certainty. In our own retrospective study, we found that AIs caused elevated testosterone levels (22.6 ± 15.9 nmol/L [range, 3.5-44.1 nmol/L], 650 ± 458 ng/dL [range, 99.3-1270 ng/dL] in early-pubertal boys; and 40.1 ± 10.5 nmol/L [range, 27.9-55.7 nmol/L], 1156 ± 302 ng/dL [range, 803-1605 ng/dL] in late-pubertal boys); increased hematocrits; and increased acne in late-pubertal boys [4].

In a new retrospective report [5] from Turkey, AI therapy in normal-weight adolescent boys (2 with gynecomastia, 17 with GH deficiency, 3 with rapid pubertal progression, and 1 with constitutional delay of growth and puberty; 6 treated with AIs alone and 17 with AIs and GH; and age > 9 years with BA < 14 years) was associated with changes in cardiac morphology and function including significantly higher (albeit not necessarily abnormal) left ventricular (LV) mass, LV mass index, LV posterior-wall diameter, and intraventricular-septal diameter (with positive correlations to serum testosterone levels [25% of which were >34.7 nmol/L; >1000 ng/dL] and hemoglobin concentrations). There was also higher myocardial systolic-wave velocity, compared to matched control groups treated with GH alone and untreated healthy boys [5]. Last, there have been no long-term studies of fertility in men previously treated with AIs during adolescence. Only one study [6] examined sperm health in 11 adolescents/young adult males with a mean age of 18.1 years previously treated with either anastrozole and GH or GH alone (last dose[s] on average, 29 months previously, 4 still receiving GH, and 7 discontinued GH 14.4 ± 4 months previously). Mean sperm concentrations and motility were comparable among the 3 groups (within the adult male range). However, the percent of normal sperm was low in all 3 groups compared to adult males, suggesting either inclusion of the wrong-aged control group or a laboratory issue across groups.

In summary, I have previously asked whether AIs are safe and effective as growth-augmenting agents 3 times in the past [6-8], but, even 16 years later, there are still no definitive answers, although maybe we are a little closer thanks to Zegarra et al. Thus, we should exert restraint in the “routine” use of AIs (especially monotherapy) and perform further research regarding their long-term efficacy and safety.

## Abbreviations

AI: aromatase inhibitor
BA: bone age
GH: growth hormone
ISS: idiopathic short stature
NAH: near-adult height
PAH: predicted adult height
